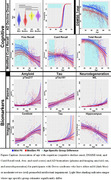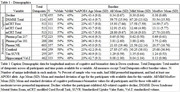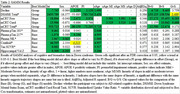# Mild premorbid intellectual impairment as a resilience factor in Down syndrome associated Alzheimer's disease

**DOI:** 10.1002/alz70856_099392

**Published:** 2025-12-24

**Authors:** James Tyler Kennedy

**Affiliations:** ^1^ Washington University in St. Louis School of Medicine, St. Louis, MO, USA

## Abstract

**Background:**

People with Down syndrome (DS) have a wide range of lifelong intellectual impairment. Triplication of the APP allele results in greater prevalence of Alzheimer's disease (AD) dementia in people with DS, typically in their 50s. Previous cross‐sectional examinations demonstrated that degree of premorbid intellectual impairment (PII) did not affect decline using linear/quadratic analyses. However, longitudinal analyses have not been performed. We analyzed longitudinal data from the Alzheimer Biomarker Consortium‐Down Syndrome (ABC‐DS) using generalized additive mixed‐models (GAMMs) to assess whether PII influences cognitive and biomarker trajectories associated with AD.

**Method:**

Individuals with Down syndrome (DS) enrolled in the longitudinal ABC‐DS study (*N* ≤ 455), were assessed for PII‐related differences in cognitive and biomarker age trajectories. GAMM models allowing offsets and slopes to differ between people with mild or moderate/severe PII were compared to submodels omitting offset/slope differences. We examined the timing of AD related cognitive decline using the modified Cued Recall Task (mCRT), and total score on the Down Syndrome Mental Status Exam (DSMSE) to test for cognitive trajectory differences. In addition, we evaluated if PII influenced plasma and neuroimaging markers of amyloid, tau, and neurodegeneration. All models included sex, APOE4 status, and karyotype as parametric effects and site/individual as random effects. Cognitive models used age at baseline and time since last test taken to account for potential practice effects.

**Result:**

Participant and visit numbers varied by measure, with 455 participants evaluated over 1178 data points. Models with PII group/slope differences were significant only for cognitive performance. Mild PII was associated with significantly later age of cognitive decline (55.2 years old vs 52.6 years old). PII influenced mCRT slope when separately examining free and cued recall scores, with mild PII's having a steeper free recall slope countered by a mild PII‐specific nonmonotonic, initially increasing, cued slope. Slope differences by PII cancel out when summed in the CRT total. Differences in PII did not significantly affect amyloid, tau, or neurodegeneration biomarker trajectories.

**Conclusion:**

Cognitive decline timing and slope differ depending on PII level, but AD biomarker pathology does not. If cognition is used for staging or endpoints in clinical trials, then PII should be considered.